# Effect of Non-thermal Atmospheric Plasma on Viability and Histamine-Producing Activity of Psychotrophic Bacteria in Mackerel Fillets

**DOI:** 10.3389/fmicb.2021.653597

**Published:** 2021-07-27

**Authors:** Marcello Trevisani, Chiara Cevoli, Luigi Ragni, Matilde Cecchini, Annachiara Berardinelli

**Affiliations:** ^1^Department of Veterinary Medical Science, Alma Mater Studiorum – University of Bologna, Bologna, Italy; ^2^Department of Agricultural and Food Sciences, Alma Mater Studiorum – University of Bologna, Bologna, Italy; ^3^Department of Industrial Engineering, University of Trento, Trento, Italy; ^4^Centre Agriculture Food Environment, University of Trento, Trento, Italy

**Keywords:** non-thermal atmospheric plasma, sodium dodecyl sulfate, histamine, *Morganella psychrotolerans*, *Photobacterium phosphoreum*, mackerel (*Scomber scombrus*), food safety

## Abstract

Non-thermal atmospheric plasma (NTAP) has gained attention as a decontamination and shelf-life extension technology. In this study its effect on psychrotrophic histamine-producing bacteria (HPB) and histamine formation in fish stored at 0–5°C was evaluated. Mackerel filets were artificially inoculated with *Morganella psychrotolerans* and *Photobacterium phosphoreum* and exposed to NTAP to evaluate its effect on their viability and the histidine decarboxylase (HDC) activity in broth cultures and the accumulation of histamine in fish samples, stored on melting ice or at fridge temperature (5°C). NTAP treatment was made under wet conditions for 30 min, using a dielectric barrier discharge (DBD) reactor. The voltage output was characterized by a peak-to-peak value of 13.8 kV (fundamental frequency around 12.7 KHz). This treatment resulted in a significant reduction of the number of *M. psychrotolerans* and *P. phosphoreum* (≈3 log cfu/cm^2^) on skin samples that have been prewashed with surfactant (SDS) or SDS and lactic acid. A marked reduction of their histamine-producing potential was also observed in HDC broth incubated at either 20 or 5°C. Lower accumulation of histamine was observed in NTAP-treated mackerel filets that have been inoculated with *M. psychrotolerans* or *P. phosphoreum* and pre-washed with either normal saline or SDS solution (0.05% w/v) and stored at 5°C for 10 days. Mean histamine level in treated and control groups for the samples inoculated with either *M. psychrotolerans* or *P. phosphoreum* (≈5 log cfu/g) varied from 7 to 32 and from 49 to 66 μg/g, respectively. No synergistic effect of SDS was observed in the challenge test on meat samples. Any detectable amount of histamine was produced in the meat samples held at melting ice temperature (0–2°C) for 7 days. The effects of NTAP on the quality properties of mackerel’s filets were negligible, whereas its effect on the psychrotrophic HPB might be useful when time and environmental conditions are challenging for the cool-keeping capacity throughout the transport/storage period.

## Introduction

Histamine is a biogenic amine and can be produced during processing and/or storage in fish, usually by the action of spoilage bacteria. The intoxication by histamine occurs after the consumption of fish and fish products containing histamine at concentrations higher than 500 ppm (Gonzaga et al., 2009; Tao et al., 2011). Hazard characterization studies identified 50 mg of histamine per meal as the dose where either adverse effects were not noted (NOAEL) or the estimate of additional risk (lower confidence level) is low, but this dosage level will not apply to individuals with a specific sensitivity to histamine and would not apply to children (FAO/WHO, 2013).

Histamine poisoning is the most common cause of human foodborne illness due to the consumption of fish products. According to the European Food Safety Authority and the European Centre for Disease Prevention and Control, 599 histamine food poisoning outbreaks were notified in the European Union (EU) during the period 2010–17 and levels of more than 3,000 ppm have been recorded in fish products implicated in outbreaks of histamine poisoning (European Food Safety Authority (EFSA), 2017; European Food Safety Authority (EFSA) and European Centre for Disease Prevention and Control (ECDC), 2018). Histamine accumulates in the tissues due to the enzymatic activity of bacteria that have contaminated the fish meat and reaches the highest concentrations in fish containing high levels of free histidine. Therefore, the intoxication is most often associated with the consumption of fish belonging to the *Scombridae* and *Scomberesocidae* families (tuna, mackerel, bonito, and jack) and some non-scombroid fish, including mahi-mahi, sardines, pilchards, anchovies, herring, marlin, and blue fin. The number and activity of Histamine-Producing Bacteria (HPB) is directly related to the probability of histamine level exceeding the microbiological criteria for the products placed on the market (Hungerford, 2010; Tao et al., 2011). The majority of HPB that have been isolated from implicated fish are Gram-negative. The psychrotrophic species that are able to produce toxic concentrations of histamine at 0–5°C, such as *Morganella psychrotolerans* (Emborg et al., 2006) and *Photobacterium phosphoreum* (Kanki et al., 2004), appear to be important in fish stored under refrigeration and mild temperature abuse (FAO/WHO, 2013). Cooling and then keeping the chill temperature of the fish is considered to be the main factor impacting the growth and/or survival of histidine-decarboxylase bacteria and consequently of histamine accumulation during storage and transport (United States Department of Health and Human Services (HHS), Food and Drug Administration (FDA), and Applied Nutrition Center for Food Safety (CFSAN), 2020). Once histidine decarboxylase (HDC) is produced, it can continue to produce histamine, even though bacterial growth has been prevented by cooling to 4°C (Kanki et al., 2007). The HDC activity is correlated with the levels of contamination and the HDC potential of bacteria. Small fishes such as mackerels are stored on-board without bleeding or gutting and are chilled to achieve a temperature close to that of melting ice. The storage can be in fish boxes or tubs with ice, also in mixture with seawater or freshwater. In order to maintain the cold chain re-icing and removal of melted ice through drainage hole of the containers are periodically made, but the cooling-keeping process could be challenged during transport and storage (EFSA Biohaz Panel et al., 2020).

Non-thermal plasma is one of the most promising minimal processing methods for the food industry (Bourke et al., 2017; López et al., 2019; Sonawane and Sonar Patil, 2020). However, up until now there are limited studies that report the application and effect of non-thermal plasma on processed seafood (Kulawik and Kumar Tiwari, 2018; Zhao et al., 2019; Dityanawarman et al., 2020; Andoni et al., 2021; Ekonomou and Boziaris, 2021). Non-thermal Atmospheric Plasma (NTAP), also called Cold Plasma, is generated under atmospheric conditions at temperatures of 30–60°C requiring low energy (Misra et al., 2011). Although the exact mechanism of bactericidal action is yet to be elucidated, it is known that NTAP is a source of multiple chemically reactive species, including reactive oxygen (ROS) and reactive nitrogen species (RNS), with a high bactericidal potency (Ziuzina et al., 2015b; Liao et al., 2016; Mai-Prochnow et al., 2016; López et al., 2019). Previous studies have indicated that cells in planktonic state show a higher sensitiveness toward NTAP than cells within biofilms (Jahid et al., 2014; Ziuzina et al., 2015a; López et al., 2019). In this respect, the use of a surfactant like sodium dodecyl sulfate (SDS) might increase the sanitation efficacy of NTAP. A synergistic effect of low pH and NTAP-derived reactive species on microbial inactivation has been also proposed (López et al., 2019) and a strong bactericidal action was demonstrated on *Listeria monocytogenes* and verotoxin-producing *Escherichia coli* when NTAP was applied in combination with SDS and lactic acid to decontaminate leafy vegetables (Berardinelli et al., 2016; Trevisani et al., 2017).

The objective of this work was to investigate the effect of NTAP and the synergic activity of SDS and lactic acid against the potential of histamine production of *M. psychrotolerans* and *P. phosphoreum* in order to explore its possible use in the processing of fresh fish.

## Materials and Methods

### Samples

Fresh mackerels (*Scomber scombrus*) were purchased in two supermarkets at different times (six different batches). The fishes (*n* = 12) were carried in insulated boxes and frozen within 1 h. Fishes were kept at −18°C for 7 days to reduce *P. phosphoreum* that can be part of the microbial background flora (Guldager et al., 1998; Emborg et al., 2002). After 7 days they were de-frozen in a cool room at 5 ± 1°C for 24 h. Surface disinfection by exposure to germicidal UV-light (254 nm, 25 w) for 30 min was also used to reduce the presence of background bacteria.

### Strains Used for the Inoculum

Five strains of *M. psychrotolerans* isolated from different samples of *Clupeids pilchardus*, *Engraulis encrasicolus*, *Clupea harengus* and *Thunnus albacares* (Costanza et al., 2013) and four strains of *P. phosphoreum* isolated from different samples of *Thunnus albacares* (FAO area 57) were used. Their ability to produce histamine was determined by inoculating the isolates in HDC broth, which was formulated with Tryptone Soy Broth (Oxoid, Basingstoke, United Kingdom), 1% l-histidine, 2% NaCl and 0.0005% pyridoxal 5-phosphate (Sigma-Aldrich, Milano, Italy) (TSBH+) and measuring with a biosensor the amount of histamine after incubation at 20°C (*M. psychrotolerans*) and 5°C (*P. phosphoreum*) for 2 and 4 days, respectively (Kanki et al., 2004). The strains have also been tested by PCR for the reference gene *vasD* (*M. psychrotolerans*) (Podeur et al., 2015) and *gyrB* (*P. phosphoreum*) (Macé et al., 2013) and for the HDC–encoding genes (De Las Rivas et al., 2005).

### Growth of Bacteria and Inoculum

Bacteria were grown in TSBH+ at 20°C (*M. psychrotolerans*) and 15°C (*P. phosphoreum*) for 2 and 4 days, respectively. Subcultures of each strain at a cell density of ≈10^8^ cfu/ml (40% transmittance at 540 nm) were mixed and used for the inoculum, which was made either on the skin or on meat samples. Spot-inoculation of the skin of entire fishes was aimed at simulating natural skin contamination and the effect of NTAP, SDS and lactic acid on the viability and histamine-producing ability that was assessed using *in vitro* test (cultures in HDC broth). With this aim entire mackerels were laid on trays and the suspensions of bacteria (either *M. psychrotolerans* or *P. phosphoreum*) (20 μl, ≈6 log cfu) were pipetted on nine different spots at a minimum distance from each other of 2.5 cm. The fish skin black lines just below the lateral line were used to mark them. All operations were done in a laminar flow hood. The contaminated fishes were then covered with an aluminum foil. The raised edges of the tray prevented it from touching the samples. In order to evaluate histamine production on fish meat stored at chilling temperature (on melting ice or at 5°C) a subsequent challenge test was made, using 5-g meat samples (1 cm thick) including the skin that were cut with sterile blades from mackerels, put in sterile weighting vessels and contaminated with ≈5.5 log cfu/g. The inoculated samples, either entire fish or meat, were held at refrigeration temperature (5 ± 1°C) overnight to allow the bacterial attachment.

### Challenge Tests

After overnight storage the mackerel’s skin samples (≈5 cm^2^) were excised with a sterile scalpel and put into weighing vessels. Not-inoculated skin samples were analyzed to detect the background HPB bacteria. The inoculated meat and skin samples were submerged in water solutions containing SDS (0.05% w/v) or in normal saline (NaCl 0.85% w/v). The effect of lactic acid (LA, 2% w/v) in combination with SDS (0.05% w/v) was also assessed on the skin samples. All reagents were purchased from Sigma-Aldrich (Milano, Italy). The preliminary washing was performed at room temperature (20–25°C) for 5 min. During this period the containers were tilted repeatedly for 5 s to increase the washing effect. After this step samples were rinsed with 18 ml of sterile deionized water to remove the residue of SDS and LA, as well as the not attached bacteria. NTAP treatment was carried out under wet conditions and at temperature of 33 ± 1°C, as previously described by Berardinelli et al. (2016). In brief, the weighting vessels with the inoculated samples were put in a plastic hermetic chamber (135 mm × 220 mm × 178 mm) housing a Dielectric Barrier Discharge (DBD) plasma source; a fan mounted above the couple of parallel electrodes was used to direct the plasma species against the sample. The vessels were filled with deionized water to a height of 0.6 cm. The distance between the fluid and the electrodes was about 2 cm. Voltage at the electrodes was produced by high voltage (HV) transformers and power switching transistors supplied by a stabilized DC power supply (Elektro-Automatik GmbH & Co., KG, EA-PS 2042-06B). Treatment parameters were fixed at 19.15 V and 3.15 ± 0.5 A for 30 min and air was used as working gas. Voltage output had a like-shaped sinusoidal waveform; during measurement its peak-to-peak value was around 13.8 kV with a fundamental frequency of oscillation around 12.7 KHz. Control samples, inoculated and washed with normal saline, were also rinsed and left for 30 min immersed in water. To ensure result reproducibility, each experiment had three replicates (skin/meat samples, 5 cm^2^/g) and was repeated two times (meat samples) or three times (skin samples) in different days using samples from different fish batches. pH of the water solutions (SDS, SDS+LA and normal saline) was measured before and immediately after the NTAP treatment. The meat samples were stored after the treatments at melting ice temperature (0–2°C) for 7 days (*n* = 6, two lots, three repetitions) and at fridge temperature (5 ± 1°C) for 10 days (*n* = 6, two lots, three repetitions). The effect of NTAP on planktonic (freely suspended) bacteria diluted in normal saline, with or without SDS, was also evaluated (*n* = 3 repetitions). Microbial suspensions (10 ml) of either *M. psychrotolerans* and *P. phosphoreum* were put in small weighing vessels and treated with NTAP as described before. The numbers of the target bacteria were counted before and after the treatment.

### Microbiological Assessments

Skin samples (5 cm^2^; ≈5 g) were homogenized in TSBH+ (1:10 dilution). Psychotropic Aerobic Viable Count (PAVC) and luminous colonies (LC) were determined by spread plating of appropriate dilutions on pre-chilled plates of Marine Agar (MA, 0.5% peptone, 0.3% yeast extract, 0.3% glycerol, agar 1.5% in micro filtrated aged sea water). Plates were incubated at 5°C and PAVC were counted after 5 days. LC were also counted in a dark room. Two colonies from MA plates of the highest dilution showing growth were collected and purified on Trypticase Soy Agar (Oxoid) supplemented with 2.0% NaCl. Then isolates were analyzed by PCR for the reference gene *gyrB* (Macé et al., 2013) to determine whether they belonged to the *P. phosphoreum* species. *M. psychrotolerans* was counted instead by spread plating the sample homogenates on MacConkey agar. Plates were incubated at 25°C for 2 days and the lactose negative colonies were counted. Five colonies picked from plates containing 10–50 cfu were transferred on Niven agar plates (0.5% Tryptone (Oxoid), 0.5% of Yeast extract (Oxoid), 0.5% NaCl (Sigma), 2% L-histidine (Sigma), 0.1% CaCO3 (Sigma), 0.006% bromocresol purple (Sigma), and 2% agar (Oxoid) (pH 5.3) (Mavromatis and Quantick, 2002). Two light purple colonies corresponding to the highest dilution showing growth were picked, streaked on trypticase soy agar (Oxoid) to obtain pure cultures and tested with the PCR assay for the reference gene *vasD* of *M. psychrotolerans* (Podeur et al., 2015). Incubation at ≈2°C (in insulated boxes with melting ice) for 7 days was used for assessing viability after NTAP treatment of freely suspended bacteria.

### Histamine Measurement in Cultures and in Meat

Skin samples (5 cm^2^) inoculated with either *M. psychrotolerans* or *P. phosphoreum* were homogenized in TSBH+ (dilution 1:10) and incubated at 20 ± 1°C and 5 ± 1°C for 1 and 5 days, respectively. The low temperature was used to inhibit the growth of mesophilic microorganisms that could prevail (Dalgaard et al., 1997). The same procedure was used for the cultures of the strains in order to assess their histamine producing potential. Meat samples (5 g) were homogenized (dilution 1:10 in phosphate buffer saline (PBS). Cultures and fish sample homogenates were sterilized at 121°C for 15 min. Then filtration by filter paper (Whatman 1004 110, Fisher Scientific, Italy) was used to remove debris. The histamine was measured with a histamine biosensor as described by Trevisani et al. (2019). Briefly, the reduction current mediated by ferrocenium ions (HCFe) was measured with an amperometer connected to a personal computer (Autolab PGSTAT 10, Methrom EcoChemie, NL) and a carbon screen-printed carbon electrode (SPCE) modified with diamine oxidase (DAO) and peroxidase (HRP). The applied potential was −0.025 mV (vs. Ag/AgCl) and measurements (mA) were taken after 120 s.

### Effect of NTAP on Quality Properties of Fish

**FIGURE 1:**
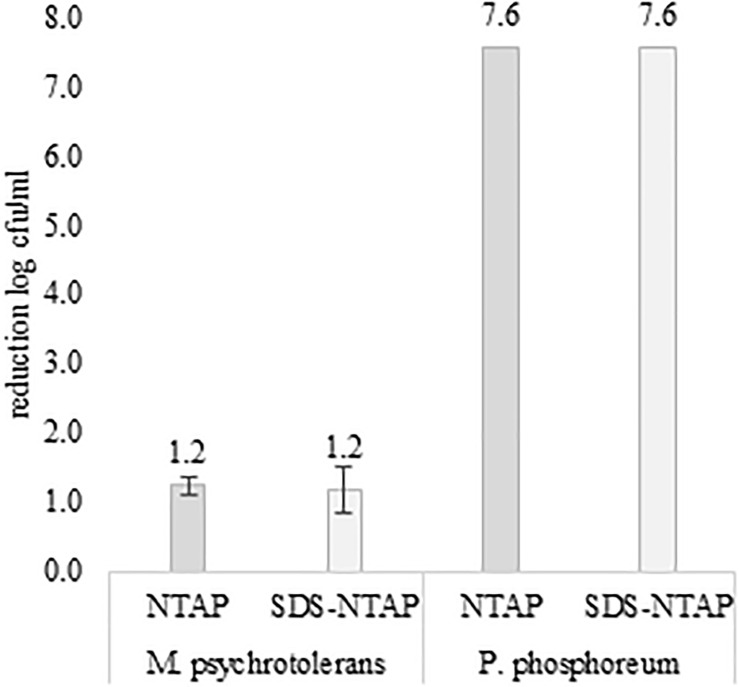
Effect of Not-Thermal Atmospheric Plasma and SDS (0.05% w/v) on *M. psychrotolerans* and *P. phosphoreum*. Counts after incubation at 2–4°C for 7 days.

Quality properties were evaluated by a trained sensory panel on 12 fishes before the treatment, immediately after the treatment (SDS+NTP, SDS+LA+NTP and NaCl) and at one, 2 and 5 days of storage. The samples were placed in a polystyrene box covered with ice flakes and stored at 4°C, up to 5 days. For the sensory evaluation, a panel of five members evaluated the fishes according to the Quality Index Method (QIM) (Özyurt et al., 2009). This sensory scale is based on the freshness quality grading system for Atlantic mackerel (*Scomber scombrus*) developed by Andrade et al. (1997). The QIM is based on the evaluation of appropriate sensory attributes of the raw fish. Demerit score ranging from 0 to 1, 2 or 3 depending on the different sensory attributes are assigned (Table 1). To obtain the quality index, the scores for all characteristics are then summed to give an overall sensory score (Botta, 1995). The scale gives zero score for absolutely fresh fish and increasing total demerit points during fish deterioration. The basic parameters of this scheme are: surface appearance; stiffness and flesh firmness; clarity of cornea, clouding of the pupil; color and smell of the gills. Color measurements of the skin were conducted by means of a Minolta ChromaMeter CR-400 reflectance colorimeter (Minolta, Milan, Italy). The acquisitions were performed on two different fish zones: the abdomen characterized by a white color and the dorsal area characterized by a dark color (Figure 1). For each acquisition, an average value of three measurements was calculated. The CIELab system L^∗^, a^∗^ and b^∗^ was considered (CIE, 1976) and the Chroma values were calculated as *C*^∗^=a2*+b2*.

**TABLE 1:** Quality Index Method (QIM) scheme for sensory evaluation.

	**Parameter**	**Characteristic**	**Demerit points**
**General appearance**	Surface appearance	Strong blue, translucent slime	0
		Loss of bright colors, pale golden tinge, slime slightly cloudy	1
		Golden tinge over all body, milky/yellowish slime	2
	Flesh firmness	Very stiff and firm	0
		Firm	1
		Fairly soft	2
		Soft, flaccid	3
	Stiffness	Very tense	0
		Tense	1
		Moderately tense	2
		Flaccid	3
**Eyes**	Clarity (cornea)	Clear, translucent	0
		Less translucent	1
		Cloudy	2
	Pupil	Black shiny	0
		Slightly cloudy	1
		Cloudy	2
	Cornea	Protuberant	0
		Convex	1
		Flat	2
		Sunken	3
**Gills**	Color	Uniformly red with blood, slime translucent	0
		Brownish with slime	1
		Dark brown, abundant slime	2
	Smell	Fresh, seaweedy	0
		Neutral	1
		Fish	2
		Ammonia	3
Range of the total demerit points			(0–20)

### Statistical Methods

Significant differences between the means of the quality parameters were evaluated by the analysis of variance (ANOVA with *Post hoc* Tukey HSD). Two-way ANOVA with replication was performed to assess the effectiveness of NTAP and its synergy with SDS/LA on the different batches of mackerels. Welch *T* test was used to analyze differences between groups where the homogeneity of variance assumption was not met. Statistical elaboration was made by Matlab R2018a.

## Results

### Effect of NTAP on Histamine-Producing Bacteria

**FIGURE 2:**
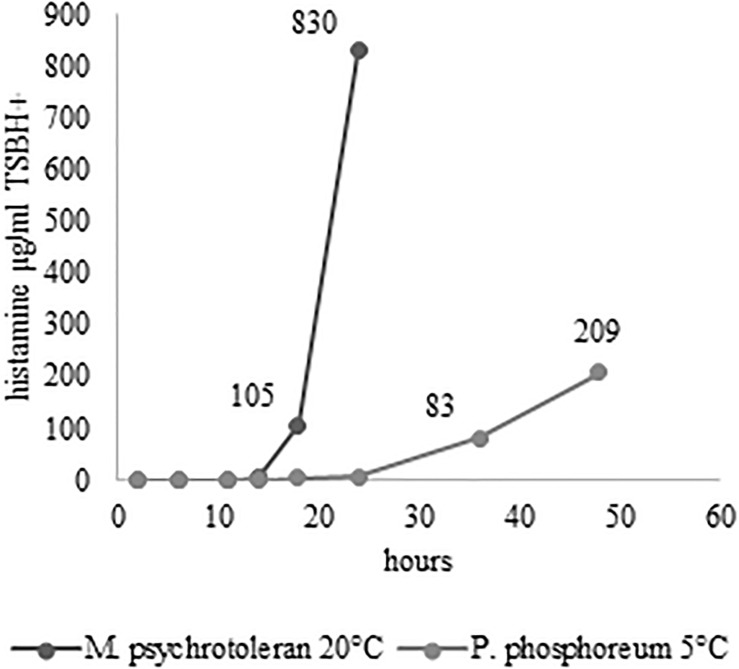
Histamine concentration in TSBH+ at 20°C for 24 h **(A)** and 5°C for 48 h **(B)**.

After 30 min of plasma exposure, the number of *P. phosphoreum* in normal saline, with or without SDS, decreased form 7.56 log cfu/ml to zero. The results were reproduced three times. A much lower antimicrobial efficacy of plasma exposure was observed with the *M. psychrotolerans* suspensions (Figure 1). No difference was observed when comparing microbial suspensions in normal saline and in SDS solutions. Temperature and availability of high concentration of free histidine (1% in TSBH+) influenced the level of histamine accumulated in the test *in-vitro* (Figure 2).

**FIGURE 3:**
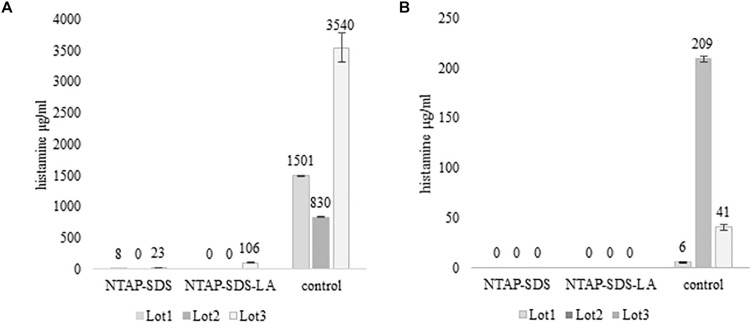
Activity of HPB at different temperature and time. Histamine produced in TSBH+. Samples not treated with NTAP and inoculated with either *M. psychrotolerans* (Mp) or *P. phosphoreum* (Pp) and incubated at 20°C and 5°C, respectively.

The challenge tests carried out with the skin samples demonstrated that NTAP can reduce the numbers and the histamine-producing potential of *M. psychrotolerans* and *P. phosphoreum* in association with an SDS pre-washing step (Table 2 and Figure 3), while lactic acid had no synergic effect. The inhibitory effect of NTAP and SDS on the viability of the target bacteria was particularly relevant with the samples incubated at 20°C, which is close to the optimum temperature for the growth of *M. psychrotolerans*, but it was significant even with the samples incubated at 5°C (*p* < 0.001). It has to be underlined that *M. psychrotolerans* and *P. phosphoreum* were detected in almost all samples independently from the inoculum and therefore, the measurements made in the challenge tests were relative to the sum of the inoculated and indigenous HPB. The concentrations of histamine produced in HDC broth at different times in two samples inoculated with similar concentration of either *M. psychrotolerans* or *P. phosphoreum* is shown in Figure 3. It is indicative of the histamine-producing potential of these bacteria in the *in-vitro* test by using similar inoculum (6 log cfu). Approximately 100 μg/ml of histamine was produced by *M. psychrotolerans* after 18 h at 20°C and similar amount (≈82 μg/ml) was produced after 36 h at 5°C by the *P. phosphoreum.*

**TABLE 2:** Inactivation of *M. psychrotolerans* and *P. phosphoreum* by non-thermal plasma associated with SDS and Lactic acid.

	**BM**	**IN**		**NTAP pre-washing**	**30 min LA+SDS**	**30 min SDS**	**No normal saline**
*M. psychrotolerans*	≈6	5.92	On skin	Lot 1	2.72	3.52	6.59
	≈2	5.87		Lot 2	0.95*	1.78	5.13
	≈4	5.89		Lot 3	3.11	2.22	5.18
				mean ± *sd*	2.26 ± *1.10*^a^	2.51 ± *0.942*^a^	5.64 ± *0.74*^b^
	ne	5.32	Meat	Lot 4	ne	0.95*	1.72
	ne	5.32		Lot 5	ne	2.65	3.57
				mean ± *sd*		1.33 ± *1.53*^a^	2.64 ± *1.13*^b^
*P. phosphoreum*	≈6	5.53	On skin	Lot 1	2.77	3.11	6.89
	≈5	5.63		Lot 2	3.03	3.49	5.98
	nd	4.82		Lot 3	2.59	3.37	4.84
				mean ± *sd*	2.79 ± *0.43*^a^	3.32 ± *0.28*^a^	5.90 ± *0.91*^b^
	ne	5.59	Meat	Lot 4	ne	2.80	3.49
	ne	5.59		Lot 5	ne	4.28	4.53
				mean ± *sd*		3.54 ± *0.86*^a^	4.01 ± *0.60*^b^

*Values are counts of bacteria (log cfu/g) identified as *M. psychrotolerans* or *P. phosphoreum* on the basis of phenotypic and genotypic characteristics. Means with a different letter in their superscript are significantly different at the 0.01 level, according to the Welch *T* test.*

BM, background microflora; IN, inoculum.

*When not detected, a value just below the limit of detection (equal to 0.9 log cfu) was assumed for the mean calculation; ne, not evaluated; nd, not detected.

**FIGURE 4:**
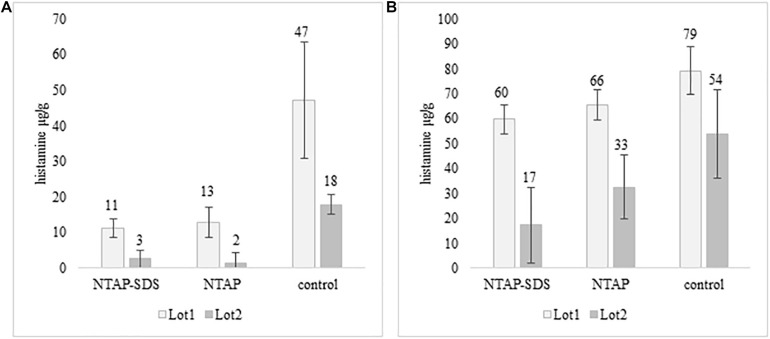
Histamine concentration (μg/g) in fish meat inoculated with *M. psychrotolerans* **(A)** and *P. phosphoreum* **(B)** after 10 days at 5°C.

Histamine was not detected in fish samples incubated at melting ice temperature for 1 week, either NTAP treated and control samples, while it was detected in those stored in fridge at 5°C for 10 days (Figure 4). The effect of NTAP on histamine production was more relevant in the samples inoculated with *M. psychrotolerans* (*p* < 0.001) leading to histamine concentrations that were approximately five times lower than in controls (i.e., 7 vs. 33 mean log cfu/g). No differences were observed in the NTAP treated samples prewashed or not with SDS. In the samples inoculated with *P. phosphoreum* the concentration of histamine was higher than in previous group and the mean difference between NTAP treated and controls was 22 μg/g (*p* < 0.01), precisely it was 28 and 17 μg/g in the samples prewashed or not with SDS. The differences observed as a consequence of pre-washing with SDS were not significant (*p* = 0.13). Inhibition of the microbial viability was also observed (Table 2), although differences between NTAP treated and controls were small (0.5–1.3 log cfu/g).

### Effect of NTAP on Quality Properties of Fish

Results of sensory evaluation, in terms of means of QIM demerit points, are shown in Table 3. Quality descriptors were related to the visual appearance of whole fish (QIM-WF), eyes (QIM-E), gills (QIM-G) and to the sum of total demerit points (QIM-tot). Demerit points (QIM-tot) increased (passing from 0 to 3.72 ± 0.21) as function of storage time, following a linear trend (R^2^ from 0.948 to 0.984), as reported for horse and Atlantic mackerel stored in ice, by Andrade et al. (1997). No differences were reported between treated and control samples, within the same storage time, confirming that the NTP does not induce sensory modifications. Concerning the color evaluation, L^∗^ and C^∗^ values are reported in Table 4. For both fish zones, significant differences were not observed during the storage for the C^∗^ parameter, while a slight significant increase in lightness was reported, for the dorsal zone, between the NTAP treated samples before and after the treatment.

**TABLE 3:** Quality index results (mean and standard deviation) over the storage time.

	**Storage (days)**	**QIM-tot**		**QIM-WF**		**QIM-E**		**QIM-G**	
Control	b	0.0	(0.0)	0.0	(0.0)	0.0	(0.0)	0.0	(0.0)
	a	0.0	(0.0)	0.0	(0.0)	0.0	(0.0)	0.0	(0.0)
	1	1.2	(2.6)	0.3	(0.7)	0.5	(1.1)	0.3	(0.7)
	2	2.1	(2.2)	0.7	(0.7)	0.8	(1.2)	0.7	(0.9)
	5	4.0	(2.1)	1.5	(1.3)	1.5	(1.0)	1.0	(1.0)
LA+SDS+NTAP	b	0.0	(0.0)	0.0	(0.0)	0.0	(0.0)	0.0	(0.0)
	a	0.0	(0.0)	0.0	(0.0)	0.0	(0.0)	0.0	(0.0)
	1	0.8	(1.9)	0.3	(0.7)	0.3	(0.7)	0.2	(0.4)
	2	2.2	(2.5)	0.7	(0.7)	0.8	(1.2)	0.7	(0.9)
	5	3.5	(1.7)	1.2	(1.1)	1.3	(0.7)	1.0	(1.0)
SDS+NTAP	b	0.0	(0.0)	0.0	(0.0)	0.0	(0.0)	0.0	(0.0)
	a	0.0	(0.0)	0.0	(0.0)	0.0	(0.0)	0.0	(0.0)
	1	1.0	(1.8)	0.3	(0.7)	0.3	(0.7)	0.3	(0.5)
	2	2.3	(1.9)	1.0	(0.8)	0.8	(1.2)	0.5	(0.8)
	5	3.7	(0.9)	1.0	(1.2)	1.5	(0.5)	1.2	(0.9)

*b, before the treatment; a, after the treatment; WF, whole fish; E, eyes; G, gills.*

**TABLE 4:** Color results.

	**Storage time (days)**	**Dorsal zone**				**Abdominal zone**			
		L*		C*		L*		C*	
Control	b	30.07	(2.73)^a^	2.58	(0.72)^a^	81.35	(8.81)^a^	7.82	(2.48)^a^
	a	32.58	(4.92)^a,b^	3.42	(1.59)^a^	82.36	(9.52)^a^	6.35	(2.22)^a^
	1	40.35	(5.67)^b,c^	4.29	(0.83)^a^	83.92	(6.26)^a^	6.04	(0.85)^a^
	2	43.71	(3.93)^c^	4.20	(0.95)^a^	83.80	(4.61)^a^	6.91	(2.45)^a^
	5	40.45	(5.85)^b,c^	3.69	(1.31)^a^	83.06	(3.30)^a^	5.39	(0.64)^a^
LA+SDS+NTAP	b	32.82	(4.78)^a^	3.15	(0.72)^a^	79.95	(3.67)^a^	6.30	(1.49)^a^
	a	40.23	(6.23)^b^	3.97	(0.44)^a^	83.52	(2.67)^a^	6.90	(1.50)^a^
	1	43.84	(4.80)^b^	4.83	(1.12)^a^	80.68	(6.01)^a^	6.19	(1.04)^a^
	2	41.34	(4.10)^b^	4.37	(1.10)^a^	79.28	(2.86)^a^	5.47	(0.73)^a^
	5	45.53	(5.10)^b^	4.38	(1.49)^a^	80.93	(1.27)^a^	7.06	(0.76)^a^
SDS+NTAP	b	30.58	(3.33)^a^	3.21	(0.62)^a^	82.68	(4.91)^a^	4.78	(2.03)^a^
	a	38.78	(4.79)^b^	3.57	(0.59)^a^	81.05	(7.30)^a^	5.88	(1.50)^a^
	1	44.93	(7.37)^b^	5.07	(1.83)^a^	85.57	(3.04)^a^	4.83	(1.25)^a^
	2	43.16	(6.99)^b^	4.95	(2.25)^a^	81.46	(2.39)^a^	6.27	(1.44)^a^
	5	44.05	(7.16)^b^	4.43	(1.04)^a^	82.99	(3.60)^a^	6.30	(2.12)^a^

*a, after the treatment; b, before the treatment.*

*Different superscript letters, within the same treatment, show significant difference (*p*-level <0.05).*

### Effect of NTAP and Residues of Sanitizers on pH

The pH of the distilled water used for NTAP treatments changed from 6.7 to 6.0 when the fish samples have been pre-washed with SDS, while remained at pH 6.1 when they have been pre-washed with LA and SDS. The pH of water used for the inoculated control samples pre-washed with normal saline was 6.7.

## Discussion

A major goal of this study was to evaluate the effect of NTAP on psychrotrophic bacteria that were associated with many incidents caused by histamine in fish and the suitability of NTAP treatment as a decontamination technique for mackerels. The experimental data demonstrated that NTAP can significantly reduce the microbial load of *M. psychrotolerans* and *P. phosphoreum* on mackerels’ skin and affect significantly their histamine-producing potential. The effect of NTAP on muscle can be affected by the buffer capability and microstructure of the proteins (Abe, 2000) that counteract the oxidative species and protect bacteria. The greater effect demonstrated using the *in-vitro* tests was nevertheless most probably related to the temperature and growth rate of the target bacteria, which have their optimum in cultures at 26–27°C (*M. psychrotolerans*) and 15–20°C (*P. phosphoreum*) (Budsberg et al., 2003; Emborg and Dalgaard, 2008) as well as to the concentration of free histidine and the decarboxylase activity of bacteria. With regard to the free histidine content of mackerels it has seasonal variations and average values between 0.20 and 0.45 g/100 g (Osako et al., 2004; FAO/WHO, 2013), while it was 1% in TSBH+. The *in vitro* model can be useful for the evaluation of NTAP activity, but the tests made using refrigerated mackerel meat have demonstrated that the treatment can be useful only for fish that are exposed to a temperature abuse and if manipulations facilitate the contamination of meat. It was found that mackerel meat samples stored at 5°C did not accumulate histamine to levels greater than 50–70 mg/kg during up to 10 days of storage when the initial level of *P. phosphoreum* was ≈3.5 – 4.5 log cfu/g, but the time to accumulate ≈82 μg/ml in TSBH+ was 36 h when the initial level was ≈ 6.0 log cfu/g. Even if enzymatic methods are specific, other biogenic amines, such as cadaverine and tyramine and especially putrescine, can interfere with histamine biosensors that are based on the DAO activity (Trevisani et al., 2017). The measured levels of histamine in fish meat could be overestimated for the presence of these diamines. Nevertheless, these values are correlated with the reduction in the numbers of *M. psychrotolerans* (1.3 log cfu/g) and *P. phosphoreum* (0.5 log cfu/g) and therefore indicative of the antimicrobial activity of NTAP treatment.

Previous studies have indicated that the presence of a critical pH of the solution, i.e., pH ≈4.7 influences the bactericidal effect by low-temperature plasma when bacteria suspensions are diluted in distilled water (Ikawa et al., 2010). They reported that acidification by plasma exposure is caused by the dissolution of nitrogen oxides generated from ambient air by the plasma and that the pH values evaluated from the NO concentration, is in good agreement with those directly measured by a pH meter. The acidity without plasma exposure, however, was ineffective and the bacterial inactivation of the target Gram- bacteria in their study arises only from the combined effect of acidity and plasma exposure. Their hypothesis was that at low pH superoxide anion radicals generated by the plasma source superoxide anion radicals are converted to hydroperoxy radicals (HOO•), which can penetrate the cell membrane and damage intercellular components.

The inhibitory effects of atmospheric CP in addition to the use of a surfactant and lactic acid on *Listeria monocytogenes*, and *E. coli* O157:H7 in red chicory was studied by Trevisani et al. (2017). They found that pre-washing red chicory with SDS before NTAP treatment reduced *E. coli* O157:H7 counts by 2.89 log CFU/g and pre-washing with lactic acid + SDS gave a much higher VTEC counts reduction of 4.78 log CFU/g. They also observed that after the treatments with (LA + SDS) 15 min + ACP 15 min and SDS 10 min + ACP 15 min, the pH values of water solutions used to immerge the leaves were 3.23 ± 0.12 and 3.32 ± 0.17, respectively. In the present study, SDS did not appear to have an additional effect on the viability of *M. psychrotolerans* that are suspended in saline solution and could not be observed in the test with *P. phosphoreum*, where NTAP alone reduced their number from 7.56 to below the detection limit. When bacteria are on food surface the activity of SDS could be most probably related to the detachment of bacteria and this effect could be different on rough or smooth hydrophobic surfaces. The plasma generated reactive oxygen and nitrogen species (RONS) can directly affect microbial cells or interact with the medium (buffer, growth media, or water) and eventually destroy microbial cells rather than damage food tissues (i.e., through oxidative stress) when the threshold level of the oxidative stress harmful to the tissues is higher than that harmful to microbial cells (Bhawana et al., 2020). Surfactants may promote the detachment and dispersion of bacteria form the surface of vegetables when they are not internalized, thus enhancing NTAP effect on microorganisms, but this effect might be easier on the surface of intact leaves (Ziuzina et al., 2015b) or the smooth surface of mackerels’ skin than on muscles.

In the present study different factors influenced the results of the challenge tests. The pH of water remained above 6.0 after the NTAP treatments even in the samples pre-washed with LA most probably as a consequence of the buffering capacity of the proteins of muscle tissue. This occurred also with the “skin samples” because a thin layer of muscle remained attached to the skin when it was excised from the fishes and it could have LA ineffective.

Differences in the temperature and in the substrate are other factors that influenced the rate of histamine production in the test made to evaluate the effect of treatments on the HPB.

Even the surfactant activity of SDS can produce different results when the washing solution are in contact with smooth surfaces like mackerel skin or on the fibrillar structure of the meat.

Ziuzina et al. (2015b) observed that the presence of organic matter in the bacterial suspension might present a protective effect against the action of RONS on bacterial cells and hypothesized that NTAP generated reactive species could not penetrate through complex biofilm matrices. The lower prevalence of bacteria that are exposed to the action of the oxidative species generated by plasma, as well as the low temperature, might explain the weak differences observed between the fish meat treated and control samples. At the melting ice temperature histamine was not produced in either control and treated samples.

The preliminary results reported in the present study would need further trials with cold plasma discharge systems capable of treating entire fishes, because in real conditions it would be the bacteria surviving to the eventual NTAP treatment made using bigger cold plasma discharge systems that produce histamine after they penetrate the natural barriers (skin, gut) in consequence of manipulations and post-mortem degradation of the tissues.

The effects of NTAP on the qualitative aspects of fresh fish were analyzed in some recent reviews (Kulawik and Kumar Tiwari, 2018; Olatunde and Benjakul, 2018). They reported that NTAP treatment induces oxidative changes of treated fish and seafood, but this does not necessary lead to decrease in sensory quality or shelf-life of the final products. In a study on mackerel filets Albertos et al. (2017) observed that NTAP (generated using a DBD plasma system operating at 50 Hz, 70–80 kV, 1–5 min) can reduce the numbers of psychrotrophic lactic acid bacteria and *Pseudomonas* up to 2 log cfu/g and that the treatment did not affect the proximate composition and color, but it induced an increase in lipid oxidation parameters, such as peroxide value and conjugated dienes, while TBARS analysis did not show significant differences. Chiper et al. (2011) reported that cold plasma generated in Ar/CO2 gas at 15 Hz, 11 kV in gas plasma can reduce the number of *Photobacterium phosphoreum* in cold smoked salmon of less than 3 log cfu/g in 2 min.

In the present study the sensory evaluation results obtained after treatment of whole mackerels suggest that the NTAP does not induce appreciable modifications after storage at 4°C for 5 days. Only a slight increase in lightness was observed, as reported also by Albertos et al. (2017) on mackerel filets treated by a DBD plasma system. This change was probably due to oxidation of hemoproteins (Kulawik and Kumar Tiwari, 2018).

In conclusion, NTAP shows potential for use as a decontamination technology in the processing of mackerels. The treatment of fishes before long distance transportations may contribute to safety and extend their shelf life. NTAP treatment might be especially useful when time and environmental conditions are challenging for the cool-keeping capacity throughout the transport/storage period.

## Data Availability Statement

The raw data supporting the conclusions of this article will be made available by the authors, without undue reservation.

## Author Contributions

MT, AB, and CC conceived the study. MT and MC made the bacteriological and chemical analyses. AB and CC made the quality and sensorial tests. MT wrote the manuscript and all authors contributed with their review.

## Conflict of Interest

The authors declare that the research was conducted in the absence of any commercial or financial relationships that could be construed as a potential conflict of interest.

## Publisher’s Note

All claims expressed in this article are solely those of the authors and do not necessarily represent those of their affiliated organizations, or those of the publisher, the editors and the reviewers. Any product that may be evaluated in this article, or claim that may be made by its manufacturer, is not guaranteed or endorsed by the publisher.
